# European consensus on essential steps of Minimally Invasive Ivor Lewis and McKeown Esophagectomy through Delphi methodology

**DOI:** 10.1007/s00464-021-08304-5

**Published:** 2021-02-19

**Authors:** Yassin Eddahchouri, Frans van Workum, Frits J. H. van den Wildenberg, Mark I. van Berge Henegouwen, Fatih Polat, Harry van Goor, MIE Delphi Collaboration, M. Asif Chaudry, E. Cheong, F. Daams, M. J. van Det, C. Gutschow, J. Heisterkamp, R. Van Hillegersberg, A. Hölscher, E. A. Kouwenhoven, M. D. P. Luyer, I. S. Martijnse, P. Nafteux, G. A. P. Nieuwenhuijzen, M. Nilsson, P. Pattyn, D. L. van der Peet, J. V. Räsänen, J. P. Ruurda, P. Schneider, W. Schröder, H. van Veer, B. P. L. Wijnhoven, Jean-Pierre E. N. Pierie, Bastiaan R. Klarenbeek, Suzanne S. Gisbertz, Camiel Rosman

**Affiliations:** 1 Department of Surgery, Radboud University Medical Center, 618, PO Box 9101, 6500 HB Nijmegen, The Netherlands; 2 Department of Surgery, Amsterdam UMC, Cancer Center Amsterdam, University of Amsterdam, Amsterdam, The Netherlands; 3 Department of Surgery, Canisius-Wilhelmina Ziekenhuis, Nijmegen, The Netherlands; 4 Department of Surgery, Medical Center Leeuwarden, Leeuwarden, The Netherlands; 5 Centrum voor Opleiding en Onderwijs Wenckebach, University Medical Center Groningen, Groningen, The Netherlands

**Keywords:** Upper GI, Consensus, Minimally invasive surgery, Esophagectomy, Essential steps

## Abstract

**Background:**

Minimally invasive esophagectomy (MIE) is a complex and technically demanding procedure with a long learning curve, which is associated with increased morbidity and mortality. To master MIE, training in essential steps is crucial. Yet, no consensus on essential steps of MIE is available. The aim of this study was to achieve expert consensus on essential steps in Ivor Lewis and McKeown MIE through Delphi methodology.

**Methods:**

Based on expert opinion and peer-reviewed literature, essential steps were defined for Ivor Lewis (IL) and McKeown (McK) MIE. In a round table discussion, experts finalized the lists of steps and an online Delphi questionnaire was sent to an international expert panel (7 European countries) of minimally invasive upper GI surgeons. Based on replies and comments, steps were adjusted and rephrased and sent in iterative fashion until consensus was achieved.

**Results:**

Two Delphi rounds were conducted and response rates were 74% (23 out of 31 experts) for the first and 81% (27 out of 33 experts) for the second round. Consensus was achieved on 106 essential steps for both the IL and McK approach. Cronbach’s alpha in the first round was 0.78 (IL) and 0.78 (McK) and in the second round 0.92 (IL) and 0.88 (McK).

**Conclusions:**

Consensus among European experts was achieved on essential surgical steps for both Ivor Lewis and McKeown minimally invasive esophagectomy.

**Supplementary Information:**

The online version of this article (doi:10.1007/s00464-021-08304-5) contains supplementary material, which is available to authorized users.

Esophageal cancer is the seventh most common cancer worldwide (572,000 cases) and the incidence is increasing [1]. The cornerstone of curative treatment of patients with locally advanced disease consists of neoadjuvant therapy followed by surgical resection. Esophagectomy is a highly complex procedure and morbidity and mortality rates up to 50% and 8% are reported, respectively [2]. The use of minimally esophagectomy (MIE) is gaining popularity [3] since it is associated with a lower complication rate and shorter hospital stay than open resection [4–6]. Long-term survival after esophagectomy depends on multiple patient and disease-related factors, but also hospital and surgeon volume have shown to affect postoperative outcome [7–10]. Moreover, extensive surgical learning curve effects of MIE on morbidity and mortality have been described [11–13]. This shows that surgical proficiency may play an important role in the outcome of surgery and shortening the learning curve could be beneficial for patient outcomes after introduction of a new surgical procedure. In MIE, several fellowship programs and courses aim to improve surgical proficiency and shortening the learning curve. However, surgical techniques are heterogeneous and essential steps of the procedure have not been established, which complicates teaching of a standardized and effective form of MIE. In addition, a consensus on the essential steps of MIE can be a foundation for a widely accepted evidence-based and structured way of training and assessment of surgical technique, which could aid in quality assurance, surgical learning and reducing learning associated morbidity.

Therefore, the primary objective of this study was to achieve international expert consensus on essential steps for both Ivor Lewis (IL) and McKeown (McK) MIE (Fig. 1) by using the Delphi methodology. Since IL and McK are the most preferred MIE approaches [3], both were incorporated in this study.

**Fig. 1:**
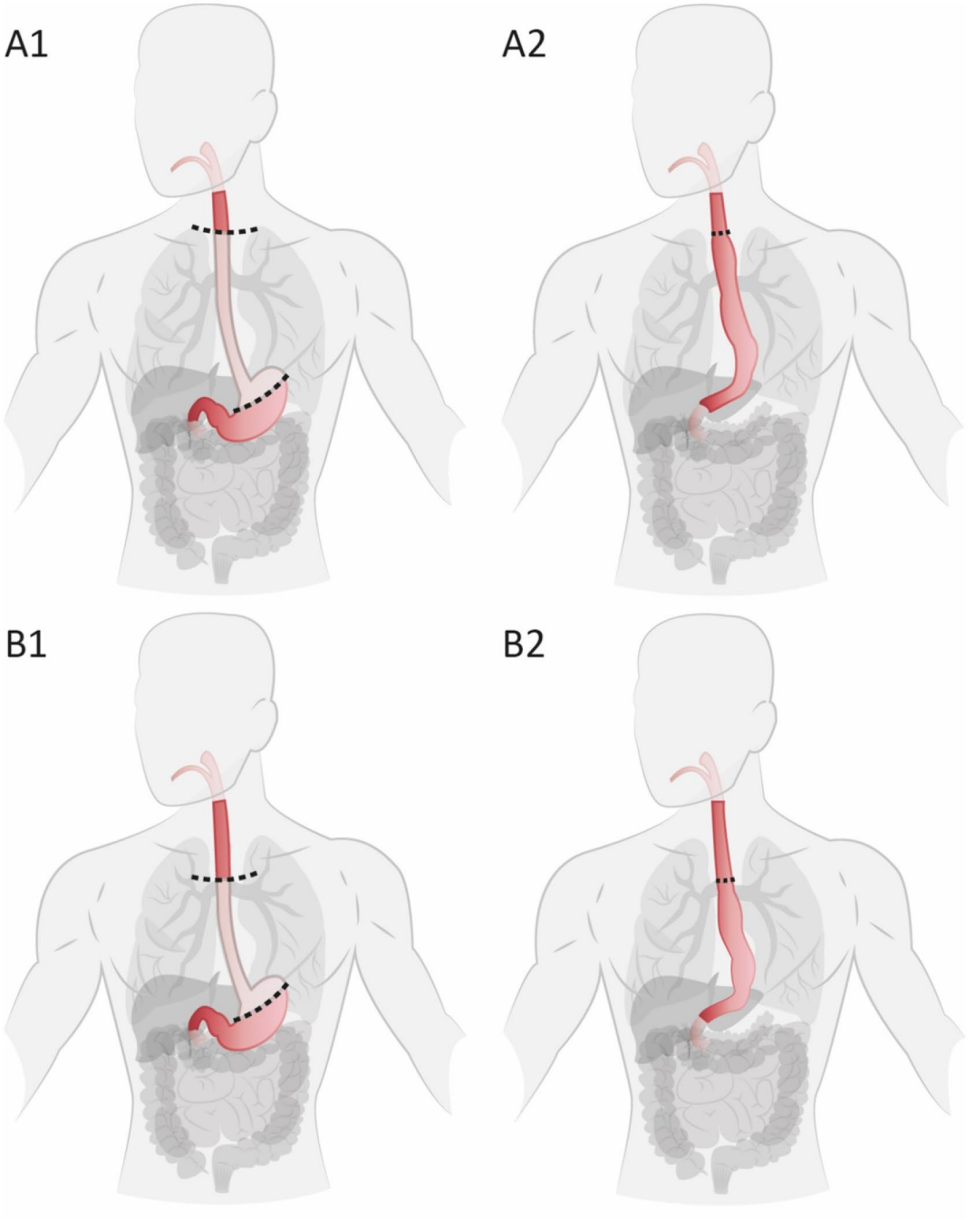
McKeown and Ivor Lewis esophagectomy. McKeown esophagus and cardia resection (**A1**) and final location of the anastomosis and gastric tube (**A2**) and Ivor Lewis resection (**B1**) and final location (**B2**). Incisions (e.g., neck incision and mini-thoracotomy) are not shown

## Methods

### Round table meetings

Led by peer-reviewed literature [14], three practicing surgeons from two high-volume hospitals experienced in thoracolaparoscopic esophagectomies (an average of 100 MIE performed so far), accompanied by one expert in surgical education, determined and defined consecutive steps required to complete MIE. The meeting was recorded to transcribe the steps, which were iteratively sent to the three surgeons for verification and refinement until the surgeons approved the version individually. This final list was used in the Delphi rounds.

### Delphi methodology

The Delphi methodology was used to achieve consensus on the essential steps of both IL and McK MIE (Fig. 1) and has been widely used in determining essential steps of other surgical procedures [15–18]. It is a process in which experts express their ideas using a questionnaire [19, 20]. Based on the responses and comments, items are adjusted, removed or added, and then resent for another round. This iterative process is ended when consensus is achieved.

### Expert panel

An international panel of practicing MIE surgeons was invited to participate in the Delphi rounds. Experts were selected based on surgical experience and involvement in training and education of surgical residents. A minimum of 100 esophagectomies and at least 3 years of experience in total MIE was required for participation. Based on expertise, involvement in research and education, we invited all members of the European Minimally Invasive Oesophagectomy (MIO) Think Tank as well as the majority of the Dutch high-volume centers. A total of 36 surgeons were invited to participate in the first round of this study. Experts were contacted by a personal invitation email, in which the aim of this study was elaborated. Then the survey was sent, followed by a personal reminder three to four weeks thereafter. Only surgeons that actively declined participation or those that did not meet inclusion criteria were not invited for next rounds.

### Ivor Lewis vs. McKeown

Since IL and McK resection are the most common MIE approaches performed [3], an individual list of essential steps was constructed for both procedures. The two lists contained several anastomotic techniques (i.e., hand-sewn end-to-end (E/E), stapled side-to-side (S/S) and stapled end-to-side (E/S) anastomosis for the IL approach and hand-sewn E/S, hand-sewn E/E, stapled E/S and stapled S/S for the McK approach). All participants received both lists and were asked to rate the MIE approach(es) and anastomotic technique(s) they regularly practiced. An anastomotic technique was excluded when rated by less than five participants. Additional procedures (i.e., nasogastric tube and jejunostomy placement) were incorporated as well.

### Delphi round one

An online questionnaire and database system [www.castoredc.com] was used to send out the Delphi questionnaire and to collect the data and comments. Panel members were asked to rate the importance of each step in MIE on a Likert-type scale; 1–5 (strongly disagree to strongly agree). Furthermore, they were asked to comment on their ratings and on any missing steps.

### Delphi round two

Responses and comments on round one were collected and analyzed. Based on the ratings and comments, steps were modified and resent to the same participants. The main modifications in the second round were rephrasing (“redefined”) steps and adding new (“new”) ones. A redefined step was a step which was changed regarding content, or which was split into multiple steps or vice versa. Modification of the steps was performed in two separate sessions by expert surgeons of two Dutch high-volume centers (> 75 MIE annually). If less than 80% of participants rated a step as 4 (“agree”) or 5 (“strongly agree”), the calculated percentages were presented back to the panel members as well. Steps that reached more than 80% agreement could also still be modified based on the comments and resent for another rating to improve agreement.

### Determination of consensus

Consensus among panel members was determined by using Cronbach’s alpha [21], which is a measure for how closely related the responses of the experts are. Missing datapoints were replaced by mean values. For scales used in research tools and for comparing groups, an Alpha of 0.7–0.8 is considered satisfactory [21]. A Cronbach’s alpha of > 0.7 was deemed satisfactory for the educational and research purpose of this study. Steps were included as an essential step when they were rated as 4 (agree) or 5 (strongly agree) by more than 80% of panel members. A new round was conducted when new steps were proposed by panel members, even when Alpha exceeded 0.7.

## Results

Three out of 36 surgeons did not perform total MIE and two surgeons of the remaining 33 did not meet the inclusion criteria at the time of the first invitation and were therefore excluded. In round one, 23 out of 31 (74%) experts from 17 hospitals and seven different countries responded to the questionnaire. The IL questionnaire was completed by 21 (91%) participants and the McK questionnaire by 16 (70%). Seven surgeons (30%) responded only to the IL questionnaire, two (9%) responded only to the McK questionnaire and 14 (61%) responded to both.

In the second round, a response rate of 81% was reached (27 out of 33 experts). The IL and McK questionnaires were completed by 24 (89%) and 18 (67%) respondents, respectively. Nine (33%) participants responded only to the IL questionnaire, three (11%) responded only to the McK questionnaire and 15 (56%) responded to both. Participating surgeons had a mean experience in MIE of ten years and had performed more than 300 MIEs in their career so far (Table 1).

**Table 1:** Expert characteristics

Experience	Ivor Lewis		McKeown	
	Mean	95% CI	Mean	95% CI
Surgery (years)	17.3	13.6–21.0	14.8	10.6–19.0
Esophageal surgery (years)	14.6	11.8–17.4	13.4	9.8–17.1
MIE (years)	10.0	7.5–12.4	9.9	6.6–13.2
MIEs performed so far (n)	340	248–432	335	212–457

*MIE* minimally invasive esophagectomy

In the first round Cronbach’s alpha reached 0.78 and 0.78 for IL and McK essential steps, respectively. In the second round Cronbach’s alpha reached 0.92 for IL and 0.88 for McK steps.

### Ivor Lewis steps

Due to the low incidence of the use of the IL hand-sewn E/E technique (one expert), the anastomotic steps of this technique were excluded. After the first round, 68 of the remaining 126 steps were directly included based on both the results and comments of the respondents. Forty-five steps were redefined to be resent for another rating, seven were resent without redefinition, five were excluded and 34 new steps were added. Some steps were redefined into multiple smaller steps or vice versa. For each step, the percentage of agreement and the action after round one (“redefined”, “resent”, “included” or “excluded”) are shown in detail in Online Appendix 1. In the second round, 81 steps (40 “redefined”, seven “resent” and 34 “new” steps) were sent to the participants, of which 43 were excluded and 38 were included, resulting in a total of 106 included (Table 2) and 48 excluded steps (Table 3). For all steps in the second round, the origin (“redefined”, “resent” or “new”), percentage of agreement and action after round two (“included” or “excluded”) are shown in detail in Online Appendix 2.

**Table 2:** Ivor Lewis final key step list

Included Ivor Lewis steps
Preparation for laparoscopic phase
1. Make sure prophylactic antibiotics are administered and repeated after 4–6 h
2. Insert urinary catheter
3. Position patient in supine position and position patient’s extremities
4. Create sterile field
5. Position operating team and position laparoscopy monitors
6. Position patient in reverse Trendelenburg
Abdominal access
7. Place 1st abdominal port and establish 12–15 mmHg pneumoperitoneum
8. Place additional ports under direct vision
9. Place liver retractor
Abdominal inspection
10. Perform diagnostic/staging laparoscopy
Mobilization of greater curvature
11. Create access to lesser sac through gastrocolic ligament
12. Dissect gastrocolic ligament along greater curvature just cranial of the transverse colon. (including preparation for later omentoplasty)
13. Dissect retrogastric adhesions onto the left crus
14. Complete dissection of gastrocolic ligament by dissecting from initiation site back to the pylorus/proximal duodenum
15. Dissect retrogastric adhesions along the pancreas to the lesser curvature
Mobilization of lesser curvature
16. Determine dissection site of gastrohepatic ligament. (3–4 side branches of right gastric artery/vein)
17. Open gastrohepatic ligament onto the stomach
18. Dissect gastrohepatic ligament along lesser curvature onto right bundle of the right crus
19. Make sure stomach is completely mobilized onto the diaphragm
Access to celiac trunk
20. Dissect peritoneum at the upper margin of the pancreas to create proper access to the celiac trunk
Identification and dissection of abdominal vessels
21. Identify right gastroepiploic vessels/arcade
22. Dissect left gastroepiploic artery and short gastric vessels
23. Free pedicle of right gastroepiploic artery of surrounding tissue to create more length
24. Identify right gastric artery
25. Identify common hepatic artery
26. Identify splenic artery
27. Identify left gastric artery and vein
28. Transect left gastric vein
29. Transect left gastric artery
Abdominal lymph node dissection
30. Dissect common hepatic artery nodes
31. Dissect left gastric artery nodes
32. Dissect celiac trunk nodes
33. Dissect proximal splenic artery nodes
34. Dissect left paracardial nodes
35. Dissect right paracardial nodes
Mobilization of distal esophagus in the hiatus
36. Dissect peritoneum of distal esophagus circumferentially
37. Transect phrenoesophageal ligaments
Creation of gastric tube
38. Determine where to start stapling
39. Place and fire first linear stapler
40. Successively fire other linear staplers
41. Make sure superior portion of the gastric tube and the distal portion of the cardia are properly (re)attached
42. Check for hemostasis along staple line
43. Check viability of gastric tube
Final abdominal inspection
44. Perform final abdominal inspection (e.g., hemostasis)
Removal of abdominal trocars, liver retractor and port closure
45. Remove trocars
46. Remove liver retractor
47. Close ports
Preparation for thoracoscopic phase
48. Position patient in preferred position (prone/semiprone/left-lateral/left-decubitus) and position patient’s extremities
49. Map thorax, including scapula margins
50. Create sterile field
51. Position operating team
52. Position thoracoscopy monitors
Thoracic access
53. Place 1st thoracic port
54. Insufflate CO2 up to 5–8 mmHg
55. Place additional ports under direct vision
Mobilization of thoracic esophagus
56. Dissect inferior pulmonary ligament
57. Dissect the pleura and mobilize the esophagus (right ventral side) along the pericardium to the level of the carina/azygos vein
58. Identify right main bronchus
59. Identify left main bronchus
60. Dissect the pleura alongside the azygos vein (from arcus azygos vein on to the level of the diaphragm)
Identification and dissection of thoracic vessels
61. Transect the arcus of the azygos vein
62. Dissect peri-esophageal aorta side branches and lymph vessels
Thoracic lymph node dissection
63. Dissect subcarinal lymph nodes
64. Dissect middle mediastinal paraesophageal lymph nodes
65. Dissect lower mediastinal paraesophageal lymph nodes
66. Dissect right pulmonary ligament lymph nodes
Thoracotomy and removal of specimen
67. Make sure esophagus is completely mobilized
68. Transect the esophagus
69. Pull esophagus and cardia and attached gastric tube into thoracic cavity
70. Perform a mini-thoracotomy
71. Place wound protector
72. Separate gastric tube from esophagus and cardia
73. Remove esophagus and cardia from thoracic cavity
Thoracic stapled E/S anastomosis
74. Make sure staple line of the gastric tube is still on the right/lateral side
75. Introduce and secure anvil into the esophagus
76. Open the tip of the gastric tube
77. Introduce circular stapler into gastric tube
78. Extend integrated trocar of the stapler through esophageal wall and connect stapler to anvil
79. Fire stapler
80. Inspect doughnuts
81. Dissect omental attachments to the surplus tip of the gastric tube
82. Dissect surplus tip of the gastric tube and remove tip from thoracic cavity
Thoracic stapled S/S anastomosis
83. Make sure staple line of the gastric tube is still on the right/lateral side
84. Open gastric tube on the side of the omentum, about 5 centimeters caudal to the tip
85. Introduce linear stapler into the gastric tube and into esophagus
86. Fire stapler
87. Close remaining opening
Omentoplasty
88. Perform omentoplasty at anastomotic site
Placement of drains
89. Place a chest drain
90. Position mediastinal drain
91. Place the drain trough the ventrolateral thoracic wall and secure drain to the skin
Irrigation and inspection
92. Check for hemostasis
93. Inspect recruited right lung before closing (i.e., position, rotation and trauma)
Removal of trocars and port/thoracotomy closure
94. Remove trocars
95. Close thoracotomy
96. Close remaining ports
Placement of nasogastric tube
97. Make sure nasogastric tube has been placed
98. Make sure nasogastric tube does not interfere with esophageal transection site and during tubulation of stomach
Jejunostomy placement
99. Identify ligament of Treitz
100. Identify jejunostomy site about 20–40 cm distally of ligament of Treitz
101. Identify efferent and afferent loop
102. Identify jejunostomy site on the abdominal wall
103. Perform jejunostomy
104. Secure jejunum to abdominal wall
105. Test patency of the catheter
106. Secure catheter to the skin

**Table 3:** Excluded Ivor Lewis steps

Ivor Lewis steps excluded after Delphi round 1 and 2	% agree
Preparation for laparoscopic phase	
1. Make sure preferred anesthetic devices are in place	79
2. Map abdomen	63
3. Mold vacuum mattress and evacuate air	38
Mobilization of greater curvature	
4. Identify mesocolon	63
5. Mobilize proximal duodenum until gastroduodenal artery is visible	75
6. Perform additional Kocher maneuver if needed	33
Identification and dissection of abdominal vessels	
7. Transect distal branches of the right gastric artery	75
8. Identify proper hepatic artery	63
9. Identify portal vein	46
Abdominal lymph node dissection	
10. Dissect hepatoduodenal ligament nodes	42
11. Dissect distal splenic artery nodes	42
12. Dissect splenic hilum nodes	8
13. Place clamp on chest drain tube (if al already in place and if already connected to reservoir)	48
Mobilization of distal esophagus in the hiatus	
14. Open left pleura	29
15. Open right pleura	54
Creation of gastric tube	
16. Oversew staple line	29
Mobilization of esophagus	
17. Retract right lung	50
18. Transect left and right vagus nerve	75
19. Open and dissect left pleura	38
Identification and dissection of thoracic vessels	
20. Transect right bronchial artery	42
21. Identify and dissect thoracic duct	50
Thoracic lymph node dissection.	
22. Dissect left upper paratracheal lymph nodes	25
23. Dissect right upper paratracheal lymph nodes	38
24. Dissect left lower paratracheal lymph nodes	46
25. Dissect right lower paratracheal lymph nodes	54
26. Dissect lymph nodes at aortopulmonary window	33
27. Dissect upper mediastinal paraesophageal lymph nodes	71
28. Dissect left pulmonary ligament lymph nodes	71
29. Completely clear the aorta of lymphatic tissue	63
Thoracotomy and removal of specimen	
30. Use specimen pack	41
Thoracic stapled E/S anastomosis	
31. Measure length of gastric tube	50
32. Make sure proximal esophagus is open (only necessary when transection done by stapler)(o)	56
33. Excise surplus cuff of the distal side of the proximal esophagus	50
34. Move camera to a port closer to the anastomotic site	63
35. Place additional sutures along this staple line. (tip gastric tube)	44
Thoracic stapled S/S anastomosis	
36. Measure length of gastric tube	67
37. Make sure proximal esophagus is open (only necessary when transection done by stapler)	67
38. Place two stitches on lateral sides of esophagus to pull esophagus on stapler	25
39. Dissect omental attachments to the surplus tip of the gastric tube	63
40. Dissect surplus tip of the gastric tube and remove tip from thoracic cavity	67
41. Place additional sutures along this staple line. (tip gastric tube)	50
Omentoplasty and/or pleuroplasty	
42. Perform pleuroplasty at anastomotic site. (fixation of anastomosis beneath plural flap)	50
Irrigation and inspection	
43. Irrigate thoracic cavity	38
44. Check for chyle leak	58
Placement of nasogastric tube	
45. Advance nasogastric tube past anastomosis, under direct vision if possible	75
Jejunostomy placement	
46. Position patient in Trendelenburg	50
47. Place extra anti-rotational stitches	79
Hiatal approximation	
48. Approximate hiatus	50

### McKeown steps

Due to the low incidence of the use of the stapled E/S (one expert) and stapled S/S technique (two experts), the steps of these techniques were excluded. After the first round, 64 of the remaining 116 steps were directly included in the final list of essential steps. Forty-one steps were redefined to be resent for another rating, ten were resent without redefinition, one was excluded and 37 new steps were added. The details of round one are shown in Online Appendix 3. In the second round, 87 steps (40 “redefined”, ten “resent” and 37 “new” steps) were sent to the participants. In this round, 45 steps were excluded and 42 were included, resulting in a total of 106 included (Table 4) and 46 excluded steps (Table 5). The details of round two are shown in Online Appendix 4.

**Table 4:** McKeown final key step list

Included McKeown steps
Preparation for thoracoscopic phase
1. Make sure prophylactic antibiotics are administered and repeated after 4–6 h
2. Position patient in preferred position (prone/semiprone/left-lateral/left-decubitus) and position patient’s extremities
3. Map thorax, including scapula margins
4. Create sterile field
5. Position operating team and position thoracoscopy monitors
Thoracic access
6. Place 1st thoracic port
7. Insufflate CO_2_ up to 5–8 mmHg
8. Place additional ports under direct vision
Mobilization of thoracic esophagus
9. Dissect inferior pulmonary ligament
10. Dissect the pleura and mobilize the esophagus (right, ventral side) along the pericardium to the level of the superior thoracic aperture
11. Identify right main bronchus
12. Identify left main bronchus
13. Dissect the pleura alongside the azygos vein from the level of the diaphragm to the superior thoracic aperture
14. Make sure esophagus is completely mobilized
Identification and dissection of thoracic vessels
15. Transect the arcus of the azygos vein
16. Dissect peri-esophageal aorta side branches and lymph vessels
Thoracic lymph node dissection
17. Dissect subcarinal lymph nodes
18. Dissect upper mediastinal paraesophageal lymph nodes
19. Dissect middle mediastinal paraesophageal lymph nodes
20. Dissect lower mediastinal paraesophageal lymph nodes
21. Dissect right pulmonary ligament lymph nodes
Irrigation and inspection
22. Check for hemostasis
23. Inspect recruited right lung before closing (i.e., position, rotation and trauma)
Removal of trocars and port closure
24. Remove trocars
25. Close ports
Preparation for laparoscopic phase
26. Position patient in supine position and position patients extremities
27. Create sterile field
28. Position operating team and position laparoscopy monitors
29. Position patient in reverse Trendelenburg
Abdominal access
30. Place 1st abdominal port and establish 12–15 mmHg pneumoperitoneum
31. Place additional ports under direct vision
32. Place liver retractor
Abdominal inspection
33. Perform diagnostic/staging laparoscopy
Mobilization of greater curvature
34. Create access to lesser sac through gastrocolic ligament
35. Dissect gastrocolic ligament along greater curvature just cranial of the transverse colon (including preparation for later omentoplasty)
36. Dissect retrogastric adhesions onto the left crus
37. Complete dissection of gastrocolic ligament by dissecting from initiation site back to the pylorus/proximal duodenum
38. Dissect retrogastric adhesions along the pancreas to the lesser curvature
Mobilization of lesser curvature
39. Determine dissection site of gastrohepatic ligament (3-4 side branches of right gastric artery/vein)
40. Open gastrohepatic ligament onto the stomach
41. Dissect gastrohepatic ligament along lesser curvature onto right bundle of the right crus
42. Make sure stomach is completely mobilized onto the diaphragm
Access to celiac trunk
43. Dissect peritoneum at the upper margin of the pancreas to create proper access to the celiac trunk
Identification and dissection of abdominal vessels
44. Identify right gastroepiploic vessels/arcade
45. Dissect left gastroepiploic artery and short gastric vessels
46. Free pedicle of right gastroepiploic artery of surrounding tissue to create more length
47. Identify right gastric artery
48. Identify common hepatic artery
49. Identify splenic artery
50. Identify left gastric artery and vein
51. Transect left gastric vein
52. Transect left gastric artery
Abdominal lymph node dissection
53. Dissect common hepatic artery nodes
54. Dissect left gastric artery nodes
55. Dissect celiac trunk nodes
56. Dissect proximal splenic artery nodes
57. Dissect left paracardial nodes
58. Dissect right paracardial nodes
Mobilization of distal esophagus in the hiatus
59. Dissect peritoneum of distal esophagus circumferentially
60. Transect phrenoesophageal ligaments
Final abdominal inspection
61. Perform final abdominal inspection (e.g., hemostasis)
Removal of abdominal trocars and port closure
62. Remove trocars
63. Remove liver retractor
64. Close ports
Cervical mobilization and transection of esophagus
65. Make skin incision anteriorly of the left sternocleidomastoid muscle
66. Divide subcutaneous tissue and platysma muscle
67. Retract sternocleidomastoid muscle and carotid sheath laterally
68. Retract larynx and trachea medially
69. Dissect esophagus away from trachea with preservation of left recurrent laryngeal nerve
70. Dissect esophagus circumferentially of remaining surrounding tissue
71. Make sure esophagus is completely mobilized
72. Transect the esophagus
Mini-laparotomy
73. Perform a mini-laparotomy^a^
Creation of gastric tube
74. Determine where to start stapling
75. Place and fire first linear stapler
76. Successively fire other linear staplers
77. Check for hemostasis along staple line
78. Check viability of gastric tube
Cervical introduction of gastric tube and removal of specimen
79. Attach a strand or drain or any other guiding device to the esophagus/specimen^a^
80. Pull esophagus/specimen into abdominal cavity/through mini-laparotomy^a^
81. Make sure to maintain a portion of the strand or drain in the neck^a^
82. Attach superior portion of the gastric tube to the strand or drain or any other guiding device^a^
83. Make sure superior portion of the gastric tube and the distal portion of the cardia are properly (re)attached^b^
84. Pull gastric tube into thoracic cavity until you reach cervical anastomotic site^c^
85. Remove distal esophagus and cardia^c^
86. Make sure staple line of the gastric tube is still on the right/lateral side^c^
Cervical hand-sewn E/S anastomosis
87. Make sure proximal esophagus is open (only necessary when transaction was done by stapler)
88. Create an opening in the gastric tube for the anastomosis
89. Create a sutured anastomosis
90. Dissect surplus tip of the gastric tube
Cervical hand-sewn E/E anastomosis
91. Dissect tip of the gastric tube
92. Create a sutured anastomosis
Wound closure
93. Close cervical wound
94. Close mini-laparotomy^a^
Placement of drains
95. Place a chest drain
Placement of nasogastric tube
96. Make sure nasogastric tube has been placed
97. Make sure nasogastric tube does not interfere with esophageal transection site and during tubulation of stomach
98. Advance nasogastric tube past anastomosis, under direct vision if possible
Jejunostomy placement
99. Identify ligament of Treitz
100. Identify jejunostomy site about 20-40 cm distally of ligament of Treitz
101. Identify efferent and afferent loop
102. Identify jejunostomy site on the abdominal wall
103. Perform jejunostomy
104. Secure jejunum to abdominal wall
105. Test patency of the catheter
106. Secure catheter to the skin

^a^In case of removing specimen abdominally

^b^In case of removing specimen through neck incision

^c^Both

**Table 5:** Excluded McKeown steps

McKeown steps excluded after Delphi round 1 and 2	% agree
Preparation for thoracoscopic phase	
1. Make sure preferred anesthetic devices are in place	67
2. Insert urinary catheter	72
Mobilization of thoracic esophagus	
3. Retract right lung	33
4. Transect left and right vagus nerve	67
5. Open and dissect left pleura	33
Identification and dissection of thoracic vessels	
6. Transect right bronchial artery	56
7. Identify and dissect thoracic duct	50
Thoracic lymph node dissection	
8. Dissect left upper paratracheal lymph nodes.	50
9. Dissect right upper paratracheal lymph nodes.	68
10. Dissect left lower paratracheal lymph nodes.	61
11. Dissect right lower paratracheal lymph nodes.	72
12. Dissect lymph nodes at aortopulmonary window.	50
13. Dissect left pulmonary ligament lymph nodes.	68
14. Completely clear the aorta of lymphatic tissue.	68
Irrigation and inspection	
15. Irrigate thoracic cavity	22
16. Check for chyle leak	44
Preparation for laparoscopic phase	
17. Map abdomen	61
18. Mold vacuum mattress and evacuate air	56
Mobilization of greater curvature	
19. Identify mesocolon	61
20. Mobilize proximal duodenum until gastroduodenal artery is visible	68
21. Perform Kocher maneuver	28
Identification and dissection of abdominal vessels	
22. Transect distal branches of the right gastric artery	72
23. Identify proper hepatic artery	78
24. Identify portal vein	56
Abdominal lymph node dissection	
25. Dissect hepatoduodenal ligament nodes	33
26. Dissect distal splenic artery nodes	44
27. Dissect splenic hilum nodes	6
Mobilization of distal esophagus in the hiatus	
28. Open left pleura	28
29. Open right pleura	56
Cervical mobilization and transection of esophagus	
30. Transect the omohyoid muscle	78
31. Identify the left recurrent laryngeal nerve	44
Identification and dissection of cervical vessels	
32. Identify middle thyroid vein	56
33. Identify inferior thyroid artery	72
34. Transect the inferior thyroid artery	61
35. Transect the middle thyroid vein	44
Cervical lymph node dissection	
36. Perform cervical lymphadenectomy	11
Creation of gastric tube	
37. Oversew staple line	44
Cervical introduction of gastric tube and removal of specimen	
38. Introduce gastric tube into thoracic cavity until you reach cervical anastomotic site by pulling esophagus/specimen through cervical incision	60
39. Introduce gastric tube into camera cover	62
Cervical stapled E/S anastomosis	
Cervical stapled S/S anastomosis	
Cervical hand-sewn E/S anastomosis	
40. Place additional sutures along this staple line.(tip gastric tube)	50
Cervical hand-sewn E/E anastomosis	
41. Make sure proximal esophagus is open (only necessary when transection was done by stapler)	63
Omentoplasty	
42. Perform omentoplasty at anastomotic site	50
Placement of drains	
43. Place and secure cervical drain	67
Jejunostomy placement	
44. Position patient in Trendelenburg	50
45. Place extra anti-rotational stitches	75
Hiatal approximation	
46. Approximate hiatus	67

## Discussion

This is the first study describing consensus-based essential steps of minimally invasive esophagectomy for cancer. Consensus among European MIE experts was achieved on essential surgical steps for both Ivor Lewis and McKeown. This resulted in a distinct list of essential steps with 106 steps for each approach, describing both procedures in detail.

### Strengths and limitations

One of the strengths of this study is that the adjustments after the first round were made at two separate occasions with local experts from two high-volume hospitals. A significant increase in consensus was reached after the second round, which demonstrated a high consensus rate compared to similar studies [15–17]. Despite a percentage of agreement ≥ 80 being the main perquisite for inclusion, comments have been used to refine or rephrase steps to improve consensus, even when this percentage was reached. Another strength of the study is that compared to the literature, and despite the length of the questionnaires (81–126 items each), high response rates of 74% and 81% were obtained for both the first and second round [16–18, 22, 23]. The international expert panel, greatly involved in education, with a vast experience in MIE and the high response rates make these lists likely to be internationally widely supported. To ensure the widely use and support of the future assessment tools, we incorporated multiple anastomotic techniques into the questionnaires. A limitation might be that participants were asked to rate the techniques they used “on a regular basis” which could have been interpreted differently by the participants. Due to the lack of expert input on the excluded techniques (IL hand-sewn E/E, McK stapled E/S and stapled S/S), we were not able to construct a consensus-based list of these steps. Another limitation is that in both rounds datapoints were missing (nine and seven percent for first and second round, respectively). Missing datapoints were replaced by respondents’ mean values to calculate Cronbach’s alpha. This method has been previously described in the literature [15]. Since sufficient consensus rates were already achieved, we believe, like in other studies, inclusion and exclusion of steps in round one was justified [20]. Finally, all experts in this study were European. Despite seven different countries were represented, the results of this study may not be easily translated to other countries and continents.

The lists of essential steps that were created present us with a detailed format that can be used to standardize MIE. In addition, it provides a starting point for developing procedure-specific assessment tools for both the entire as well as certain parts of the operation. Since final mastery of the procedure comes literally step-by-step, a validated assessment tool for specific parts of the procedure would facilitate specific and structured feedback for residents, fellows and surgeons. This will help to objectively evaluate and assure a surgeons’ proficiency and might potentially shorten the learning curve and, more importantly, diminish the learning associated morbidity and mortality. In bariatric surgery, patients operated by surgeons in the top quartile of skills seem less likely to develop overall complications as compared to the bottom quartile [24]. Moreover, in complex oncologic procedures technical performance among credentialed surgeons varies substantially, which is significantly associated with clinical and pathological outcomes [25]. This emphasizes the need to improve one’s surgical skills as fast and efficient as possible, especially in complex oncologic procedures like MIE. Procedure-specific assessment tools differentiate well between different skills levels and they seem to be more suitable for summative assessment than global rating scales [26]. For example, the procedure-specific assessment tool in laparoscopic cholecystectomy seemed to better differentiate between novice, intermediate and almost competent trainees than the OSATS and GOALS. Since complex procedures, like MIE, are taught to surgeons that already have surgical experience in other procedures, technical differences between novice and expert surgeons might be subtler. Therefore, a specific assessment tool may be more suitable to allow for a unique insight in the dependence between different levels of skills and outcome of surgery in minimally invasive esophagectomy.

## Conclusion

In this study, we described consensus-based essential steps of minimally invasive esophagectomy for cancer. Future perspectives include the development and validation of an assessment tool targeting essential steps associated with clinically relevant outcome parameters.

## Supplementary Information

Below is the link to the electronic supplementary material.

Electronic supplementary material 1 (DOCX 95 kb)
