# Supplementary material for: European consensus on essential steps of Minimally Invasive Ivor Lewis and McKeown Esophagectomy through Delphi methodology
Source: Surg Endosc. 2021 Feb 19;36(1):446–60. doi: 10.1007/s00464-021-08304-5 (PMC8741699; doi:10.1007/s00464-021-08304-5)
Supplement: Supplementary file 1 — Electronic supplementary material 1 (DOCX 95 kb) [file 464_2021_8304_MOESM1_ESM.docx]

**Appendix 1: Ivor Lewis round 1 details**

| **Round 1 Ivor Lewis steps** | % agree | Redefined / resent / excluded (# in **2^nd^ round survey**) | Included (# in **final** list) |
| --- | --- | --- | --- |
| **­­­­­Preparation for laparoscopic phase** |  |  |  |
| 1. Make sure nasogastric tube, endotracheal tube and central venous catheter are in place. | 81 | Redefined (1, 74) |  |
| 1. Position patient in supine position. | 100 | Redefined (4) |  |
| 1. Position legs and arms using supports. | 76 | Redefined (4) |  |
| 1. Mold vacuum mattress and evacuate air. | 38 | Excluded |  |
| 1. Map abdomen, including location of jejunostomy tube and chest drain. | 48 | Redefined (5) |  |
| 1. Create sterile field. | 91 |  | Included (4) |
| 1. Take place in between patient’s legs. | 71 | Redefined (6) |  |
| 1. Assisting operator takes place on the patient’s left side. | 57 | Redefined (6) |  |
| 1. OR assistant takes place on the patient’s right side. | 43 | Redefined (6) |  |
| 1. Position laparoscopy monitors. | 95 | Redefined (6) |  |
| 1. Position patient in reverse Trendelenburg. | 91 |  | Included (6) |
| **Chest drain placement** |  |  |  |
| 1. Place chest drain. (prevention of tension pneumothorax during laparoscopy) | 48 | Redefined (70) |  |
| **Abdominal access** |  |  |  |
| 1. Place 1^st^ abdominal port and establish 12-15 mmHg pneumoperitoneum. | 86 |  | Included (7) |
| 1. Place additional ports under direct vision. | 100 |  | Included (8) |
| **Diagnostic laparoscopy** |  |  |  |
| 1. Perform diagnostic laparoscopy. | 95 |  | Included (10) |
| **Placement of liver retractor** |  |  |  |
| 1. Introduce and position liver retractor. | 95 |  | Included (9) |
| **Mobilization of greater curvature** |  |  |  |
| 1. Identify right gastroepiploic artery/arcade. | 100 |  | Included (21) |
| 1. Create access to lesser sac through gastrocolic ligament. | 95 |  | Included (11) |
| 1. Dissect gastrocolic ligament along greater curvature just cranial of the transverse colon. (including preparation for later omentoplasty) | 95 |  | Included (12) |
| 1. Dissect short gastric vessels. | 100 | Redefined (13) |  |
| 1. Dissect retrogastric adhesions on to the left crus. | 100 |  | Included (13) |
| 1. Complete dissection of gastrocolic ligament by dissecting from initiation site back to the pylorus. | 100 | Redefined (8) |  |
| 1. Dissect retrogastric adhesions along the pancreas to the lesser curvature until you encounter the right gastroepiploic artery. | 95 | Redefined (9) |  |
| 1. Free pedicle of right gastroepiploic artery of surrounding tissue to create more length. | 86 |  | Included (23) |
| **Mobilization of lesser curvature** |  |  |  |
| 1. Determine dissection site of gastrohepatic ligament. *(3-4 side branches of right gastric artery/vein)* | 91 |  | Included (16) |
| 1. Open gastrohepatic ligament on to the stomach. | 91 |  | Included (17) |
| 1. Identify right gastric artery. | 86 |  | Included (24) |
| 1. Transect right gastric artery. | 52 | Redefined (14) |  |
| 1. Dissect gastrohepatic ligament along lesser curvature onto right bundle of the right crus. | 96 |  | Included (18) |
| 1. Dissect peritoneum from the right bundle of the right crus to the left bundle of the right crus. | 91 | Redefined (26) |  |
| 1. Transect phrenoesophageal ligaments. | 100 |  | Included (37) |
| 1. Dissect peritoneum at the level of the left gastric artery. | 80 | Redefined (12) |  |
| 1. Identify common hepatic artery. | 100 |  | Included (25) |
| 1. Identify splenic artery. | 100 |  | Included (26) |
| 1. Dissect lymph nodes at left gastric artery and common hepatic artery. | 90 | Redefined (17, 19) |  |
| 1. Identify left gastric artery and vein. | 95 |  | Included (27) |
| 1. Transect left gastric vein. | 100 |  | Included (28) |
| 1. Transect left gastric artery. | 100 |  | Included (29) |
| 1. Dissect lymph nodes along splenic artery. | 95 | Redefined (21- 23) |  |
| 1. Make sure stomach is completely mobilized on to the diaphragm. | 95 |  | Included (19) |
| **Creation of gastric tube** |  |  |  |
| 1. Retract nasogastric tube until it does not interfere with esophageal transaction site. | 86 | Redefined (75) |  |
| 1. Determine where to start stapling. | 91 |  | Included (38) |
| 1. Place and fire first linear stapler. | 95 |  | Included (39) |
| 1. Successively fire other linear staplers until cardia and fundus are separated. | 76 | Redefined (29) |  |
| 1. Check for hemostasis along staple line. | 86 |  | Included (42) |
| 1. Check viability of gastric tube. | 81 |  | Included (43) |
| 1. Stitch superior portion of the gastric tube to the distal portion of the cardia. | 62 | Redefined (30) |  |
| **Inspection of splenic region** |  |  |  |
| 1. Check for hemostasis at splenic region. | 95 | Redefined (32) |  |
| **Removal of liver retractor** |  |  |  |
| 1. Remove liver retractor. | 91 |  | Included (46) |
| **Jejunostomy placement** |  |  |  |
| 1. Position patient in Trendelenburg. | 62 | Resent (77) |  |
| 1. Identify ligament of Treitz. | 86 |  | Included (99) |
| 1. Identify jejunostomy site about 40 cm distally of ligament of Treitz. | 76 | Redefined (78) |  |
| 1. Identify efferent and afferent loop. | 86 |  | Included (101) |
| 1. Determine jejunostomy site on the abdominal wall. | 86 |  | Included (102) |
| 1. Perform jejunostomy. | 80 |  | Included (103) |
| 1. Secure jejunum to abdominal wall. | 85 |  | Included (104) |
| 1. Test patency of the catheter. | 86 |  | Included (105) |
| 1. Secure catheter to the skin. | 75 | Resent (80) |  |
| **Removal of trocars and port closure** |  |  |  |
| 1. Remove trocars. | 91 |  | Included (45) |
| 1. Close fascia and skin. | 91 |  | Included (47) |
| **Preparation for thoracoscopic phase** |  |  |  |
| 1. Position patient in prone position. | 52 | Redefined (33) |  |
| 1. Position arms in diving position. | 52 | Redefined (33) |  |
| 1. Place clamp on chest drain tube. (if al already in place and if already connected to reservoir) | 48 | Excluded |  |
| 1. Map thorax, including scapula margins. | 86 |  | Included (49) |
| 1. Create sterile field. | 90 |  | Included (50) |
| 1. Operator and assistant operator take place on the patient’s right side. | 71 | Redefined (34) |  |
| 1. OR assistant takes place on the patient’s left side. | 62 | Redefined (34) |  |
| 1. Position thoracoscopy monitors. | 90 |  | Included (52) |
| **Thoracic access** |  |  |  |
| 1. Place 1^st^ thoracic port and insufflate CO2 up to 8-10 mmHg. | 76 | Redefined (35, 36) |  |
| 1. Place additional ports under direct vision. | 100 |  | Included (55) |
| **Mobilization of esophagus** |  |  |  |
| 1. Dissect inferior pulmonary ligament. | 95 |  | Included (56) |
| 1. Dissect the pleura along the pericardium to the level of the carina. | 95 | Redefined (38) |  |
| 1. Identify right main bronchus. | 100 |  | Included (58) |
| 1. Dissect subcarinal lymph nodes. (station VII) | 86 |  | Included (59) |
| 1. Dissect the pleura on the ventral side of the esophagus. (on to the level of the azygos vein) | 91 | Redefined (38) |  |
| 1. Transect the azygos vein. | 76 | Redefined (43) |  |
| 1. Dissect the pleura in-between esophagus and the aorta, on the dorsal side of esophagus. (on to the level of the azygos vein) | 95 | Redefined (41) |  |
| 1. Mobilize the esophagus at the level of the carina or transection site. | 100 | Redefined 38) |  |
| 1. Expand mobilization of the esophagus just cranially to the level of transection. | 91 | Redefined (38) |  |
| 1. Transect the esophagus. | 95 |  | Included (68) |
| 1. Dissect peri-esophageal attachments/aorta side branches/lymph vessels. | 95 |  | Included (62) |
| 1. Paraesophageal lymph nodes are dissected en bloc during the mobilization of the esophagus. | 95 | Redefined (51-53) |  |
| 1. Make sure esophagus is completely mobilized. | 100 |  | Included (67) |
| **Mini-thoracotomy** |  |  |  |
| 1. Perform a mini-thoracotomy. | 91 |  | Included (70) |
| 1. Place wound protector. | 86 |  | Included (71) |
| **Thoracic stapled E/S anastomosis** |  |  |  |
| 1. Excise about ¾ of esophageal staple line. | 36 | Redefined (61) |  |
| 1. Introduce and secure anvil into the esophagus. | 75 | Resent (62) |  |
| 1. Excise surplus cuff of the distal side of the proximal esophagus. | 50 | Excluded |  |
| 1. Pull esophagus and cardia and attached gastric tube into thoracic cavity. | 92 |  | Included^a^ (69) |
| 1. Make sure staple line of the gastric tube is still on the right/lateral side. | 100 |  | Included (74) |
| 1. Detach gastric tube from esophagus and cardia. | 75 | Redefined (57) |  |
| 1. Remove esophagus and cardia through the wound protector. | 83 | Redefined (59) |  |
| 1. Open the tip of the gastric tube. | 83 |  | Included (76) |
| 1. Introduce circular stapler into gastric tube. | 100 |  | Included (77) |
| 1. Move camera to a port closer to the anastomotic site. | 75 | Resent (63) |  |
| 1. Extend integrated trocar of the stapler through esophageal wall and connect stapler to anvil. | 83 |  | Included (78) |
| 1. Fire stapler. | 100 |  | Included (79) |
| 1. Inspect doughnuts. | 100 |  | Included (80) |
| 1. Dissect omental attachments to the surplus tip of the gastric tube. | 83 |  | Included (81) |
| 1. Dissect surplus tip of the gastric tube (stapler) and remove tip from thoracic cavity. | 100 |  | Included (82) |
| 1. Place additional sutures along this staple line. | 67 | Resent (64) |  |
| **Thoracic stapled S/S anastomosis** |  |  |  |
| 1. Pull esophagus and cardia and attached gastric tube into thoracic cavity. | 100 |  | Included^a^ (69) |
| 1. Make sure staple line of the gastric tube is still on the right/lateral side. | 100 |  | Included (74) |
| 1. Detach gastric tube from esophagus and cardia. | 100 | Redefined (57) |  |
| 1. Remove esophagus and cardia through the wound protector. | 63 | Redefined (59) |  |
| 1. Place two stitches on lateral sides of esophagus to pull esophagus on stapler. | 25 | Excluded |  |
| 1. Excise esophageal staple line in between the two stitches. | 38 | Redefined (66) |  |
| 1. Open gastric tube on the side of the omentum, about 5 centimeters caudal to the tip. | 100 |  | Included (84) |
| 1. Introduce linear stapler into the gastric tube and into esophagus. | 100 |  | Included (85) |
| 1. Fire stapler. | 100 |  | Included (86) |
| 1. Advance nasogastric tube past anastomosis. | 75 | Redefined (76) |  |
| 1. Close remaining opening. | 100 |  | Included (87) |
| 1. Dissect omental attachments to the surplus tip of the gastric tube. | 63 | Excluded |  |
| 1. Dissect surplus tip of gastric tube (stapler) and remove tip through wound protector. | 75 | Redefined (67) |  |
| 1. Place additional sutures along this staple line. | 63 | Resent (68) |  |
| **Omentoplasty and/or pleuroplasty** |  |  |  |
| 1. Perform omentoplasty at anastomotic site. | 80 |  | Included (88) |
| 1. Perform pleuroplasty at anastomotic site. | 25 | Redefined (69) |  |
| **Mediastinal drain placement** |  |  |  |
| 1. Position mediastinal drain. | 95 |  | Included (90) |
| 1. Place the drain trough the ventrolateral thoracic wall and secure drain to the skin. | 86 |  | Included (91) |
| **Irrigation and inspection** |  |  |  |
| 1. Irrigate thoracic cavity. | 43 | Resent (71) |  |
| 1. Check for hemostasis. | 91 |  | Included (92) |
| Nasogastric tube placement |  |  |  |
| 1. Advance nasogastric tube past anastomosis if not done yet. | 76 | Redefined (76) |  |
| **Removal of trocars** |  |  |  |
| 1. Remove trocars. | 95 |  | Included (94) |
| **Insufflation of the right lung** |  |  |  |
| 1. Insufflate right lung again under direct vision. | 86 | Redefined (73) |  |
| **Port and thoracotomy closure** |  |  |  |
| 1. Close thoracotomy. | 100 |  | Included (95) |
| 1. Close remaining ports. | 95 |  | Included (96) |

^a^ 89 and 102 were combined into 1 step (69) as a result of change in order of the final list.

**Appendix 2: Ivor Lewis round 2 details**

| **Round 2 Ivor Lewis steps** | New / redefined | % agree | Included / excluded (# in **final** list) |
| --- | --- | --- | --- |
| **Preparation for laparoscopic phase** |  |  |  |
| 1. Make sure preferred anesthetic devices are in place. | Redefined | 79 | Excluded |
| 1. Make sure prophylactic antibiotics are administered and repeated after 4-6 hours. | New | 87 | Included (1) |
| 1. Insert urinary catheter. | New | 83 | Included (2) |
| 1. Position patient in supine position and position patient’s extremities. | Redefined | 87 | Included (3) |
| 1. Map abdomen. | Redefined | 63 | Excluded |
| 1. Position operating team and position laparoscopy monitors. | Redefined | 92 | Included (5) |
| **Mobilization of greater curvature** |  |  |  |
| 1. Identify mesocolon. | New | 63 | Excluded |
| 1. Complete dissection of gastrocolic ligament by dissecting from initiation site back to the pylorus/proximal duodenum. (if not done previously) | Redefined | 96 | Included (14) |
| 1. Dissect retrogastric adhesions along the pancreas to the lesser curvature. | Redefined | 100 | Included (15) |
| 1. Mobilize proximal duodenum until gastroduodenal artery is visible. | New | 75 | Excluded |
| 1. Perform addtional Kocher maneuver if needed. | New | 33 | Excluded |
| **Access to celiac trunk** |  |  |  |
| 1. Dissect peritoneum at the upper margin of the pancreas to create proper access to the celiac trunk. | Redefined | 96 | Included (20) |
| **Identification and dissection of abdominal vessels** |  |  |  |
| 1. Dissect left gastroepiploic artery and short gastric vessels. | Redefined | 96 | Included (22) |
| 1. Transect distal branches of the right gastric artery. | Redefined | 75 | Excluded |
| 1. Identify proper hepatic artery. | New | 63 | Excluded |
| 1. Identify portal vein. | New | 46 | Excluded |
| **Abdominal lymph node dissection** |  |  |  |
| 1. Dissect common hepatic artery nodes. | Redefined | 82 | Included (30) |
| 1. Dissect hepatoduodenal ligament nodes. | New | 42 | Excluded |
| 1. Dissect left gastric artery nodes. | Redefined | 100 | Included (31) |
| 1. Dissect celiac trunk nodes. | New | 96 | Included (32) |
| 1. Dissect proximal splenic artery nodes. | Redefined | 88 | Included (33) |
| 1. Dissect distal splenic artery nodes. | Redefined | 42 | Excluded |
| 1. Dissect splenic hilum nodes. | Redefined | 8 | Excluded |
| 1. Dissect left paracardial nodes. | New | 100 | Included (34) |
| 1. Dissect right paracardial nodes. | New | 100 | Included (35) |
| **Mobilization of distal esophagus in the hiatus** |  |  |  |
| 1. Dissect peritoneum of distal esophagus circumferentially. | Redefined | 92 | Included (36) |
| 1. Open left pleura. | New | 29 | Excluded |
| 1. Open right pleura. | New | 54 | Excluded |
| **Creation of gastric tube** |  |  |  |
| 1. Successively fire other linear staplers. | Redefined | 96 | Included (40) |
| 1. Make sure superior portion of the gastric tube and the distal portion of the cardia are properly (re)attached. | Redefined | 92 | Included (41) |
| 1. Oversew staple line. | New | 29 | Excluded |
| **Final abdominal inspection and Removal of abdominal trocars and port closure.** |  |  |  |
| 1. Perform final abdominal inspection. (e.g. hemostasis) | Redefined | 100 | Included (44) |
| **Preparation for thoracoscopic phase** |  |  |  |
| 1. Position patient in preferred position (prone/semiprone/left-lateral/left-decubitus) and position patient’s extremities. | Redefined | 96 | Included (48) |
| 1. Position operating team. | Redefined | 96 | Included (51) |
| **Thoracic access** |  |  |  |
| 1. Place 1^st^ thoracic port. | Redefined | 96 | Included (53) |
| 1. Insufflate CO2 up to 5-8 mmHg. | Redefined | 88 | Included (54) |
| **Mobilization of esophagus** |  |  |  |
| 1. Retract right lung. | New | 50 | Excluded |
| 1. Dissect the pleura and mobilize the esophagus (right ventral side) along the pericardium to the level of the carina/azygos vein. | Redefined | 100 | Included (57) |
| 1. Identify left main bronchus. | New | 100 | Included (59) |
| 1. Transect left and right vagus nerve. | New | 75 | Excluded |
| 1. Dissect the pleura alongside the azygos vein. (from arcus azygos vein on to the level of the diaphragm) | Redefined | 100 | Included (60) |
| 1. Open and dissect left pleura. | New | 38 | Excluded |
| **Identification and dissection of thoracic vessels** |  |  |  |
| 1. Transect the arcus of the azygos vein. | Redefined | 88 | Included (61) |
| 1. Transect right bronchial artery. | New | 42 | Excluded |
| 1. Identify and dissect thoracic duct. | New | 50 | Excluded |
| **Thoracic lymph node dissection.** |  |  |  |
| 1. Dissect left upper paratracheal lymph nodes. | New | 25 | Excluded |
| 1. Dissect right upper paratracheal lymph nodes. | New | 38 | Excluded |
| 1. Dissect left lower paratracheal lymph nodes. | New | 46 | Excluded |
| 1. Dissect right lower paratracheal lymph nodes. | New | 54 | Excluded |
| 1. Dissect lymph nodes at aortopulmonary window. | New | 33 | Excluded |
| 1. Dissect upper mediastinal paraesophageal lymph nodes. | Redefined | 71 | Excluded |
| 1. Dissect middle mediastinal paraesophageal lymph nodes. | Redefined | 96 | Included (64) |
| 1. Dissect lower mediastinal paraesophageal lymph nodes. | Redefined | 96 | Included (65) |
| 1. Dissect left pulmonary ligament lymph nodes. | New | 71 | Excluded |
| 1. Dissect right pulmonary ligament lymph nodes. | New | 92 | Included (66) |
| 1. Completely clear the aorta of lymphatic tissue. | New | 63 | Excluded |
| **Thoracotomy and removal of specimen.** |  |  |  |
| 1. Separate gastric tube from esophagus and cardia. | Redefined | 96 | Included (72) |
| 1. Use specimen pack. | New | 41 | Excluded |
| 1. Remove esophagus and cardia from thoracic cavity. | Redefined | 96 | Included (73) |
| **Thoracic stapled E/S anastomosis** |  |  |  |
| 1. Measure length of gastric tube. | New | 50 | Excluded |
| 1. Make sure proximal esophagus is open. (only necessary when transection done by stapler) | Redefined | 56 | Excluded |
| 1. Introduce and secure anvil into the esophagus. | Resent | 81 | Included (75) |
| 1. Move camera to a port closer to the anastomotic site. | Resent | 63 | Excluded |
| 1. Place additional sutures along this staple line. | Resent | 44 | Excluded |
| **Thoracic stapled S/S anastomosis** |  |  |  |
| 1. Measure length of gastric tube. | New | 67 | Excluded |
| 1. Make sure proximal esophagus is open. (only necessary when transection done by stapler) | Redefined | 67 | Excluded |
| 1. Dissect surplus tip of the gastric tube and remove tip from thoracic cavity. | Redefined | 67 | Excluded |
| 1. Place additional sutures along this staple line. | Resent | 50 | Excluded |
| **Omentoplasty and/or pleuroplasty** |  |  |  |
| 1. Perform pleuroplasty at anastomotic site. (fixation of anastomosis beneath plural flap) | Redefined | 50 | Excluded |
| **Placement of drains** |  |  |  |
| 1. Place a chest drain. | Redefined | 96 | Included (89) |
| **Irrigation and inspection and - Removal of trocars and port/thoracotomy closure.** |  |  |  |
| 1. Irrigate thoracic cavity. | Resent | 38 | Excluded |
| 1. Check for chyle leak. | New | 58 | Excluded |
| 1. Inspect recruited right lung before closing. (i.e. position/rotation, and trauma) | Redefined | 96 | Included (93) |
| **Placement of nasogastric tube** |  |  |  |
| 1. Make sure nasogastric tube has been placed. | Redefined | 90 | Included (96) |
| 1. Make sure nasogastric tube does not interfere with esophageal transection site and during tabulation of stomach. | Redefined | 80 | Included (97) |
| 1. Advance nasogastric tube past anastomosis, under direct vision if possible. | Redefined | 75 | Excluded |
| **Jejunostomy placement** |  |  |  |
| 1. Position patient in Trendelenburg. | Resent | 50 | Excluded |
| 1. Identify jejunostomy site about 20-40 cm distally of ligament of Treitz. | Redefined | 100 | Included (100) |
| 1. Place extra anti-rotational stitches. | New | 79 | Excluded |
| 1. Secure catheter to the skin. | Resent | 93 | Included (106) |
| **Hiatal approximation.** |  |  |  |
| 1. Approximate hiatus. | New | 50 | Excluded |

**Appendix 3: McKeown round 1 details**

| **Round 1 McKeown steps** | % agree | Redefined / resent / excluded (# in **2^nd^ round survey** list) | Included (# in **final** list) |
| --- | --- | --- | --- |
| **Preparation for thoracoscopic phase** |  |  |  |
| 1. Make sure nasogastric tube, endotracheal tube and central venous catheter are in place. | 94 | Redefined (1, 81) |  |
| 1. Position patient in prone position | 56 | Redefined (4) |  |
| 1. Position arms in diving position | 56 | Redefined (4) |  |
| 1. Map thorax, including scapula margins | 94 |  | Included (3) |
| 1. Create sterile field | 100 |  | Included (4) |
| 1. Operator and assistant operator take place on the patient’s right side | 69 | Redefined (5) |  |
| 1. OR assistant takes place on the patient’s left side | 56 | Redefined (5) |  |
| 1. Position laparoscopy monitors | 94 | Redefined (5) |  |
| **Thoracic access** |  |  |  |
| 1. Place 1^st^ thoracic port and insufflate CO2 up to 8-10 mmHg | 69 | Redefined (6, 7) |  |
| 1. Place additional ports under direct vision. | 100 |  | Included (8) |
| **Mobilization of esophagus** |  |  |  |
| 1. Dissect inferior pulmonary ligament. | 100 |  | Included (9) |
| 1. Dissect the pleura along the pericardium onto the level of the carina. | 100 | Redefined (9) |  |
| 1. Identify right main bronchus. | 100 |  | Included (11) |
| 1. Dissect subcarinal lymphnodes (station VII). | 81 |  | Included (17) |
| 1. Dissect the pleura cranially on the ventral side of esophagus (up to the superior thoracic aperture). | 88 | Redefined (9) |  |
| 1. Transect the azygos vein. | 100 | Redefined (14) |  |
| 1. Dissect the pleura in-between esophagus and the aorta, on the dorsal side of esophagus (up to the superior thoracic aperture). | 100 | Redefined (12) |  |
| 1. Mobilize the esophagus cranially (up to the superior thoracic aperture). | 100 | Redefined (9) |  |
| 1. Dissect peri-esophageal attachments/aorta side branches/lymph vessels. | 94 |  | Included (16) |
| 1. Paraesophageal lymph nodes are dissected en bloc during the mobilization of the esophagus. | 94 | Redefined (22 -24) |  |
| 1. Make sure esophagus is completely mobilized. | 100 |  | Included (14) |
| **Irrigation and inspection** |  |  |  |
| 1. Irrigate thoracic cavity. | 31 | Resent (28) |  |
| 1. Check for hemostasis. | 100 |  | Included (22) |
| **Removal of trocars and port closure** |  |  |  |
| 1. Remove trocars. | 100 |  | Included (24) |
| 1. Close all ports. | 88 |  | Included (25) |
| **Preparation for laparoscopic phase** |  |  |  |
| 1. Position patient in supine position. | 100 | Redefined (31) |  |
| 1. Position legs and arms using supports. | 94 | Redefined (31) |  |
| 1. Mold vacuum mattress and evacuate air. | 56 | Excluded |  |
| 1. Map abdomen, including location of jejunostomy tube and chest drain. | 63 | Redefined (32) |  |
| 1. Create sterile field. | 100 |  | Included (27) |
| 1. Take place in between patient’s legs. | 63 | Redefined (33) |  |
| 1. Assisting operator takes place on the patient’s left side. | 69 | Redefined (33) |  |
| 1. OR assistant takes place on the patient’s right side. | 50 | Redefined (33) |  |
| 1. Position laparoscopy monitors. | 100 | Redefined (33) |  |
| 1. Position patient in reverse Trendelenburg. | 94 |  | Included (29) |
| **Chest drain placement** |  |  |  |
| 1. Place chest drain (prevention of tension pneumothorax during laparoscopy). | 63 | Redefined (83) |  |
| **Abdominal access** |  |  |  |
| 1. Place 1^st^ abdominal port and establish 12-15 mmHg pneumoperitoneum. | 88 |  | Included (30) |
| 1. Place additional ports under direct vision. | 100 |  | Included (31) |
| **Diagnostic laparoscopy** |  |  |  |
| 1. Perform diagnostic laparoscopy. | 88 |  | Included (33) |
| **Placement of liver retractor** |  |  |  |
| 1. Introduce and position liver retractor. | 94 |  | Included (32) |
| **Mobilization of greater curvature** |  |  |  |
| 1. Indentify right gastroepiploic artery/arcade. | 100 |  | Included (44) |
| 1. Create access to lesser sac through gastrocolic ligament. | 88 |  | Included (34) |
| 1. Dissect gastrocolic ligament along greater curvature just cranial of the transverse colon (including preparation for later omentoplasty). | 100 |  | Included (35) |
| 1. Dissect short gastric vessels. | 100 | Redefined (40) |  |
| 1. Dissect retrogastric adhesions onto the left crus. | 100 |  | Included (36) |
| 1. Complete dissection of gastrocolic ligament by dissecting from initiation site back to the pylorus (if not done previously. | 100 | Redefined (35) |  |
| 1. Dissect retrogastric adhesions along the pancreas to the lesser curvature until you encounter the right gastroepiploic artery. | 94 | Redefined (36) |  |
| 1. Free pedicle of right gastroepiploic artery of surrounding tissue to create more length. | 88 |  | Included (46) |
| **Mobilization of lesser curvature** |  |  |  |
| 1. Determine dissection site of gastroheptic ligament *(3-4 side branches of right gastric artery/vein).* | 100 |  | Included (39) |
| 1. Open gastrohepatic ligament onto the stomach. | 94 |  | Included (40) |
| 1. Identify right gastric artery. | 94 |  | Included (47) |
| 1. Transect right gastric artery. | 69 | Redefined (41) |  |
| 1. Dissect gastrohepatic ligament along lesser curvature onto right bundle of right crus. | 100 |  | Included (41) |
| 1. Dissect peritoneum from the right bundle of the right crus to the left bundle of the right crus. | 94 | Redefined (53) |  |
| 1. Transect phrenoesophageal ligaments. | 94 |  | Included (60) |
| 1. Dissect peritoneum at the level of the left gastric artery. | 94 | Redefined (39) |  |
| 1. Identify common hepatic artery. | 100 |  | Included (48) |
| 1. Identify splenic artery. | 100 |  | Included (49) |
| 1. Dissect lymph nodes at left gastric artery and common hepatic artery. | 94 | Redefined (44, 46) |  |
| 1. Identify left gastric artery and vein. | 100 |  | Included (50) |
| 1. Transect left gastric vein. | 100 |  | Included (51) |
| 1. Transect left gastric artery. | 100 |  | Included (52) |
| 1. Dissect lymph nodes along splenic artery. | 100 | Redefined (48-50) |  |
| 1. Make sure stomach is completely mobilized onto the diaphragm. | 100 |  | Included (42) |
| **Inspection of splenic region** |  |  |  |
| 1. Check for hemostasis at the splenic region. | 100 | Redefined (56) |  |
| **Removal of liver retractor** |  |  |  |
| 1. Remove liver retractor. | 82 |  | Included (63) |
| **Jejunostomy placement** |  |  |  |
| 1. Position patient in Trendelenburg. | 63 | Resent (84) |  |
| 1. Identify ligament of Treitz. | 94 |  | Included (99) |
| 1. Identify jejunostomy site about 40 cm distally of ligament of Treitz. | 81 | Redefined (85) |  |
| 1. Identify efferent and afferent loop. | 94 |  | Included (101) |
| 1. Determine jejunostomy site on the abdominal wall. | 94 |  | Included (102) |
| 1. Perform jejunostomy. | 87 |  | Included (103) |
| 1. Secure jejunum to abdominal wall. | 93 |  | Included (104) |
| 1. Test patency of the catheter. | 94 |  | Included (105) |
| 1. Secure catheter to the skin. | 88 |  | Included (106) |
| **Removal of trocars and port closure** |  |  |  |
| 1. Remove trocars. | 81 |  | Included (62) |
| 1. Close fascia and skin. | 88 |  | Included (64) |
| **Cervical transection of esophagus** |  |  |  |
| 1. Make skin incision anteriorly of the left sternocleidomastoid muscle. | 100 |  | Included (65) |
| 1. Divide subcutaneous tissue and platysma muscle. | 100 |  | Included (80) |
| 1. Retract sternocleidomastoid muscle and carotid sheath laterally. | 100 |  | Included (67) |
| 1. Transect the omohyoid muscle. | 57 | Resent (57) |  |
| 1. Identify the left recurrent laryngeal nerve. | 57 | Resent (58) |  |
| 1. Retract larynx and trachea medially. | 86 |  | Included (68) |
| 1. Identify middle and inferior thyroid artery. | 71 | Redefined (60, 61) |  |
| 1. Transect the inferior thyroid artery. | 64 | Resent (62) |  |
| 1. Transect the middle thyroid artery and vein. | 50 | Redefined (63) |  |
| 1. Dissect esophagus away from trachea with preservation of left recurrent laryngeal nerve. | 93 |  | Included (69) |
| 1. Dissect esophagus circumferentially of remaining surrounding tissue. | 100 |  | Included (70) |
| 1. Perform cervical lymphadenectomy. | 21 | Resent (64) |  |
| 1. Make sure esophagus is completely mobilized. | 100 |  | Included (71) |
| 1. Transect the esophagus (stapler). | 7 | Redefined (59) |  |
| 1. Attach a strand or extra-large penrose drain to the distal esophagus. | 50 | Redefined (68) |  |
| **Mini-laparotomy** |  |  |  |
| 1. Perform a mini-laparotomy. | 53 | Redefined (65) |  |
| **Creation of gastric tube and removal of distal esophagus and cardia** |  |  |  |
| 1. Pull esophagus into abdominal cavity or through mini-laparotomy. | 50 | Resent (70) |  |
| 1. Make sure to maintain a portion of the strand or drain in the neck. | 80 |  | Included (81) |
| 1. Determine where to start stapling | 88 |  | Included (74) |
| 1. Place and fire first linear stapler | 88 |  | Included (75) |
| 1. Successively fire other linear staplers until cardia and fundus are separated. | 88 | Redefined (66) |  |
| 1. Remove distal esophagus and cardia. | 80 |  | Included (85) |
| 1. Check for hemostasis along staple line. | 94 |  | Included (77) |
| 1. Check viability of gastric tube. | 87 |  | Included (78) |
| 1. Introduce gastric tube into camera cover. | 60 | Resent (72) |  |
| 1. Attach superior portion of the gastric tube to the strand or drain. | 73 | Redefined (73) |  |
| 1. Pull gastric tube into thoracic cavity until you reach cervical anastomotic site. | 93 |  | Included (104) |
| 1. Make sure staple line of the gastric tube is still on the right/lateral side. | 93 |  | Included (86) |
| **Cervical hand-sewn E/S anastomosis** |  |  |  |
| 1. Excise esophageal staple line. | 46 | Redefined (74) |  |
| 1. Create an opening in the gastric tube for the anastomosis. | 100 |  | Included (88) |
| 1. Create a sutured anastomosis. | 100 |  | Included (89) |
| 1. Dissect surplus tip of the gastric tube. | 91 |  | Included (90) |
| 1. Place additional sutures along this staple line. | 36 | Resent (75) |  |
| **Cervical hand-sewn E/E anastomosis** |  |  |  |
| 1. Excise esophageal staple line. | 67 | Redefined (76) |  |
| 1. Dissect tip of the gastric tube. | 100 |  | Included (91) |
| 1. Create a sutured anastomosis. | 100 |  | Included (92) |
| **Drain placement** |  |  |  |
| 1. Place and secure cervical drain. | 73 | Resent (79) |  |
| **Nasogastric tube placement** |  |  |  |
| 1. Advance nasogastric tube past anastomosis (if not done yet). | 73 | Redefined (83) |  |
| **Cervical wound closure** |  |  |  |
| 1. Close wound. | 86 |  | Included (93) |

**Appendix 4: McKeown round 2 details**

| **Round 2 McKeown steps** | New / redefined / resent | % agree | Included / excluded (# in **final** list) |
| --- | --- | --- | --- |
| **Preparation for thoracoscopic phase** |  |  |  |
| 1. Make sure preferred anesthetic devices are in place. | Redefined | 67 | Excluded |
| 1. Make sure prophylactic antibiotics are administered and repeated after 4-6 hours. | New | 89 | Included (1) |
| 1. Insert urinary catheter. | New | 72 | Excluded |
| 1. Position patient in preferred position (prone/semiprone/left-lateral/left-decubitus) and position patient’s extremities. | Redefined | 89 | Included (2) |
| 1. Position operating team and position thoracoscopy monitors. | Redefined | 94 | Included (5) |
| **Thoracic access** |  |  |  |
| 1. Place 1^st^ thoracic port. | Redefined | 94 | Included (6) |
| 1. Insufflate CO2 up to 5-8 mmHg. | Redefined | 89 | Included (7) |
| **Mobilization of thoracic esophagus** |  |  |  |
| 1. Retract right lung. | New | 33 | Excluded |
| 1. Dissect the pleura and mobilize the esophagus (right ventral side) along the pericardium to the level of the superior thoracic aperture. | Redefined | 94 | Included (10) |
| 1. Identify left main bronchus. | New | 94 | Included (12) |
| 1. Transect left and right vagus nerve. | New | 67 | Excluded |
| 1. Dissect the pleura alongside the azygos vein from the level of the diaphragm to the superior thoracic aperture. | Redefined | 94 | Included (13) |
| 1. Open and dissect left pleura. | New | 33 | Excluded |
| **Identification and dissection of thoracic vessels** |  |  |  |
| 1. Transect the arcus of the azygos vein. | Redefined | 94 | Included (15) |
| 1. Transect right bronchial artery. | New | 56 | Excluded |
| 1. Identify and dissect thoracic duct. | New | 50 | Excluded |
| **Thoracic lymph node dissection.** |  |  |  |
| 1. Dissect left upper paratracheal lymph nodes. | New | 50 | Excluded |
| 1. Dissect right upper paratracheal lymph nodes. | New | 68 | Excluded |
| 1. Dissect left lower paratracheal lymph nodes. | New | 61 | Excluded |
| 1. Dissect right lower paratracheal lymph nodes. | New | 72 | Excluded |
| 1. Dissect lymph nodes at aortopulmonary window. | New | 50 | Excluded |
| 1. Dissect upper mediastinal paraesophageal lymph nodes. | Redefined | 83 | Included (18) |
| 1. Dissect middle mediastinal paraesophageal lymph nodes. | Redefined | 100 | Included (19) |
| 1. Dissect lower mediastinal paraesophageal lymph nodes. | Redefined | 100 | Included (20) |
| 1. Dissect left pulmonary ligament lymph nodes. | New | 68 | Excluded |
| 1. Dissect right pulmonary ligament lymph nodes. | New | 94 | Included (21) |
| 1. Completely clear the aorta of lymphatic tissue. | New | 68 | Excluded |
| **Irrigation and inspection** |  |  |  |
| 1. Irrigate thoracic cavity. | Resent | 22 | Excluded |
| 1. Check for chyle leak. | New | 44 | Excluded |
| 1. Inspect recruited right lung before closing. (i.e. position/rotation, and trauma) | New | 83 | Included (23) |
| **Preparation for laparoscopic phase** |  |  |  |
| 1. Position patient in supine position and position patient’s extremities. | Redefined | 94 | Included (26) |
| 1. Map abdomen. | Redefined | 61 | Excluded |
| 1. Position operating team and position laparoscopy monitors. | Redefined | 89 | Included (28) |
| **Mobilization of greater curvature** |  |  |  |
| 1. Identify mesocolon. | New | 61 | Excluded |
| 1. Complete dissection of gastrocolic ligament by dissecting from initiation site (1) back to the pylorus/proximal duodenum. | Redefined | 100 | Included (37) |
| 1. Dissect retrogastric adhesions along the pancreas to the lesser curvature. | Redefined | 100 | Included (38) |
| 1. Mobilize proximal duodenum until gastroduodenal artery is visible. | New | 68 | Excluded |
| 1. Perform Kocher maneuver. | New | 28 | Excluded |
| **Access to celiac trunk.** |  |  |  |
| 1. Dissect peritoneum at the upper margin of the pancreas to create proper access to the celiac trunk. | Redefined | 94 | Included (43) |
| **Identification and dissection of abdominal vessels.** |  |  |  |
| 1. Dissect left gastroepiploic artery and short gastric vessels. | Redefined | 100 | Included (45) |
| 1. Transect distal branches of the right gastric artery. | Redefined | 72 | Excluded |
| 1. Identify proper hepatic artery. | New | 78 | Excluded |
| 1. Identify portal vein. | New | 56 | Excluded |
| **Abdominal lymph node dissection** |  |  |  |
| 1. Dissect common hepatic artery nodes. | Redefined | 83 | Included (53) |
| 1. Dissect hepatoduodenal ligament nodes. | New | 33 | Excluded |
| 1. Dissect left gastric artery nodes. | Redefined | 100 | Included (54) |
| 1. Dissect celiac trunk nodes. | New | 100 | Included (55) |
| 1. Dissect proximal splenic artery nodes. | Redefined | 89 | Included (56) |
| 1. Dissect distal splenic artery nodes. | Redefined | 44 | Excluded |
| 1. Dissect splenic hilum nodes. | Redefined | 6 | Excluded |
| 1. Dissect left paracardial nodes. | New | 100 | Included (57) |
| 1. Dissect right paracardial nodes. | New | 100 | Included (58) |
| **Mobilization of distal esophagus in the hiatus** |  |  |  |
| 1. Dissect peritoneum of distal esophagus circumferentially. | Redefined | 94 | Included (59) |
| 1. Open left pleura. | New | 28 | Excluded |
| 1. Open right pleura. | New | 56 | Excluded |
| **Final abdominal inspection.** |  |  |  |
| 1. Perform final abdominal inspection. (e.g. hemostasis) | Redefined | 100 | Included (61) |
| **Cervical mobilization and transection of esophagus.** |  |  |  |
| 1. Transect the omohyoid muscle. | Resent | 78 | Excluded |
| 1. Identify the left recurrent laryngeal nerve. | Resent | 44 | Excluded |
| 1. Transect the esophagus. | Redefined | 89 | Included (72) |
| **Identificaiton and dissection of cervical vessels** |  |  |  |
| 1. Identify middle thyroid vein. | Redefined | 56 | Excluded |
| 1. Identify inferior thyroid artery. | Redefined | 72 | Excluded |
| 1. Transect the inferior thyroid artery. | Resent | 61 | Excluded |
| 1. Transect the middle thyroid vein. | Redefined | 44 | Excluded |
| **Cervical lymph node dissection.** |  |  |  |
| 1. Perform cervical lymphadenectomy. | Resent | 11 | Excluded |
| **Mini-laparotomy** |  |  |  |
| 1. Perform a mini-laparotomy. (not necessary in case of removing specimen through neck incision) | Redefined | 100 | Included (73) |
| **Creation of gastric tube** |  |  |  |
| 1. Successively fire other linear staplers. | Redefined | 83 | Included (76) |
| 1. Oversew staple line. | New | 44 | Excluded |
| **Cervical introduction of gastric tube and removal of specimen** |  |  |  |
| 1. Attach a strand or drain or any other guiding device to the esophagus/specimen. | Redefined | 100 | Included (68) |
| 1. Make sure superior portion of the gastric tube and the distal portion of the cardia are properly (re)attached. | New | 100 | Included (83) |
| 1. Pull esophagus/specimen into abdominal cavity or through mini-laparotomy. | Resent | 100 | Included (80) |
| 1. Introduce gastric tube into thoracic cavity until you reach cervical anastomotic site by pulling esophagus/specimen through cervical incision. | New | 60 | Excluded |
| 1. Introduce gastric tube into camera cover. | Resent | 62 | Excluded |
| 1. Attach superior portion of the gastric tube to the strand or drain or any other guiding device. | Redefined | 85 | Included (82) |
| **Cervical hand-sewn E/S anastomosis** |  |  |  |
| 1. Make sure proximal esophagus is open (only necessary when transaction was done by stapler). | Redefined | 83 | Included (87) |
| 1. Place additional sutures along this staple line. | Resent | 50 | Excluded |
| **Cervical hand-sewn E/E anastomosis** |  |  |  |
| 1. Make sure proximal esophagus is open (only necessary when transection was done by stapler). | Redefined | 63 | Excluded |
| **Wound closure** |  |  |  |
| 1. Close mini-laparotomy. | New | 100 | Included (94) |
| **Omentoplasty** |  |  |  |
| 1. Perform omentoplasty at anastomotic site. | New | 50 | Excluded |
| **Placement of drains** |  |  |  |
| 1. Place and secure cervical drain. | Resent | 67 | Excluded |
| 1. Pace a chest drain. | Redefined | 100 | Included (95) |
| **Placement of nasogastric tube** |  |  |  |
| 1. Make sure nasogastric tube has been placed. | Redefined | 94 | Included (96) |
| 1. Make sure nasogastric tube does not interfere with esophageal transection site and during tabulation of stomach. | New | 83 | Included (97) |
| 1. Advance nasogastric tube past anastomosis, under direct vision if possible. | Redefined | 89 | Included (98) |
| **Jejunostomy placement** |  |  |  |
| 1. Position patient in Trendelenburg. | Resent | 50 | Excluded |
| 1. Identify jejunostomy site about 20-40 cm distally of ligament of Treitz. | Redefined | 100 | Included (100) |
| 1. Place extra anti-rotational stitches. | New | 75 | Excluded |
| **Hiatal approximation** |  |  |  |
| 1. Approximate hiatus. | New | 67 | Excluded |
